# Draft Genome Sequence of *Sinomicrobium* sp. Strain PAP.21, Isolated from a Coast Sample of Papua, Indonesia

**DOI:** 10.1128/mra.01268-22

**Published:** 2023-03-21

**Authors:** Riyanti, Celine M. Zumkeller, Marius Spohn, Sanja Mihajlovic, Oliver Schwengers, Alexander Goesmann, Riviani Riviani, Maria D. N. Meinita, Till F. Schäberle, Harwoko Harwoko

**Affiliations:** a Faculty of Fisheries and Marine Science, Jenderal Soedirman University, Purwokerto, Indonesia; b Branch for Bioresources, Fraunhofer Institute for Molecular Biology and Applied Ecology (IME), Giessen, Germany; c Bioinformatics and Systems Biology, Justus Liebig University Giessen, Giessen, Germany; d German Center for Infection Research (DZIF), Partner Site Giessen-Marburg-Langen, Giessen, Germany; e Institute for Insect Biotechnology, Justus Liebig University Giessen, Giessen, Germany; f Department of Pharmacy, Faculty of Health Sciences, Jenderal Soedirman University, Purwokerto, Indonesia; Montana State University

## Abstract

*Sinomicrobium* sp. strain PAP.21 (EXT111902) was isolated from the coast of Cenderawasih Bay National Park in West Papua, Indonesia. Its genome was assembled into 151 contigs with a total size of 5.439 Mbp, enabling the prediction of its specialized metabolite production capacity.

## ANNOUNCEMENT

The phylum *Bacteroidetes* is a promising, although underexplored, bioresource for natural product discovery (1, 2). *Bacteroidetes* are among the most abundant bacteria within marine ecosystems (3), and their known bioactive natural products represent a remarkable diversity. This diversity is, e.g., exemplified by the lanthipeptide pinensin from Chitinophaga pinensis exhibiting antifungal activity (4). Expecting a positive correlation between the *Bacteroidetes* diversity and their produced chemical diversity, we aimed at accessing new strains.

In this study, *Sinomicrobium* sp. PAP.21 was isolated from marine sediment of the upper layer (5 to 10 cm) collected at a coast area of Cenderawasih Bay National Park, Papua, Indonesia (2°23′06.8″S 134°57′53.5″E). The strain was deposited in the Fraunhofer strain collection (5) under its identifier EXT111902.

The marine sediment sample was stored in a sterile plastic bag before being plated onto artificial seawater (1.5% agar, 0.01% KBr, 2.3% NaCl, 1.1% MgCl_2_ · 6 H_2_O, 0.1% CaCl_2_ · 2H_2_O, 0.1% KCl, 0.004% SrCl_2_ · 6 H_2_O, 0.4% Na_2_SO_4_, 0.02% NaHCO_3_, and 0.003% H_3_BO_3_ in H_2_O), including Escherichia coli prey, and incubated at 30°C. Grown colonies were purified on marine agar (Carl Roth GmbH; product no. CP73.1 + 1.5% agar), and a sequencing sample was prepared by growing EXT111902 aerobically in marine broth (30°C for 24 h; Carl Roth GmbH; product no. CP73.1). The cell pellet was resuspended in ATL buffer (Qiagen) containing RNase A. BashingBead lysis tubes (Zymo Research) were used for cell disruptions. DNA was isolated using QIAmp 96 DNA QIAcube high-throughput (HT) kits with the addition of proteinase K (Qiagen). Libraries for short-read sequencing were prepared using the Illumina DNA prep tagmentation kit with 500 ng DNA input and 5 cycles of indexing PCR. Library quality was evaluated (Agilent 2100 bioanalyzer) and sequenced on an Illumina NovaSeq instrument using a NovaSeq 6000 SP v1 sequencing kit with a 2 × 150 bp read length and a depth of 4.0 to 5.0 Mio reads. For sequence processing and analysis, software tools were run with default settings unless otherwise stated. The sequencing was demultiplexed (Illumina bcl2fastq, v2.19.0.316), quality checked (Fastp [6] v0.20.1), and visualized (MultiQC [7] v1.7). A total of 11.98 million paired-end reads were quality filtered (Fastp [6] v0.20.1; additional parameter “–detect_adapter_for_pe –cut_by_quality5 –cut_by_quality3 –low_complexity_filter –length_required 21 –correction”), assembled (Unicycler [8] v0.4.8), and annotated (Bakta [9] v1.5.1). The genome consists of 5,438,544 bp (coverage, 320×; *N*_50_, 141,686 bp; *L*_50_, 13) in 151 contigs and has a GC content of 44.4%. Using CheckM (v1.0.18) (10), the degree of genome completeness was determined at 99.34% with 3.9% contamination. The genome encodes 4,679 protein-coding genes, 47 tRNAs, 1 transfer-messenger RNA (tmRNA), 3 rRNAs and 6 noncoding RNAs (ncRNAs). The taxonomical rank was established using the Type Strain Genome Server (11). This process revealed Sinomicrobium oceani CGMCC 1.12145 (12) as the closest related type strain. Digital DNA-DNA hybridization (dDDH) values exceed the species delineation threshold of 70% (76.9% [d0], 92.1% [d4], and 82.3% [d6]). An average nucleotide identity (ANI) (13) value of 98.89% supports the affiliation of EXT111902 to the species *S. oceani*.

**FIG 1:**
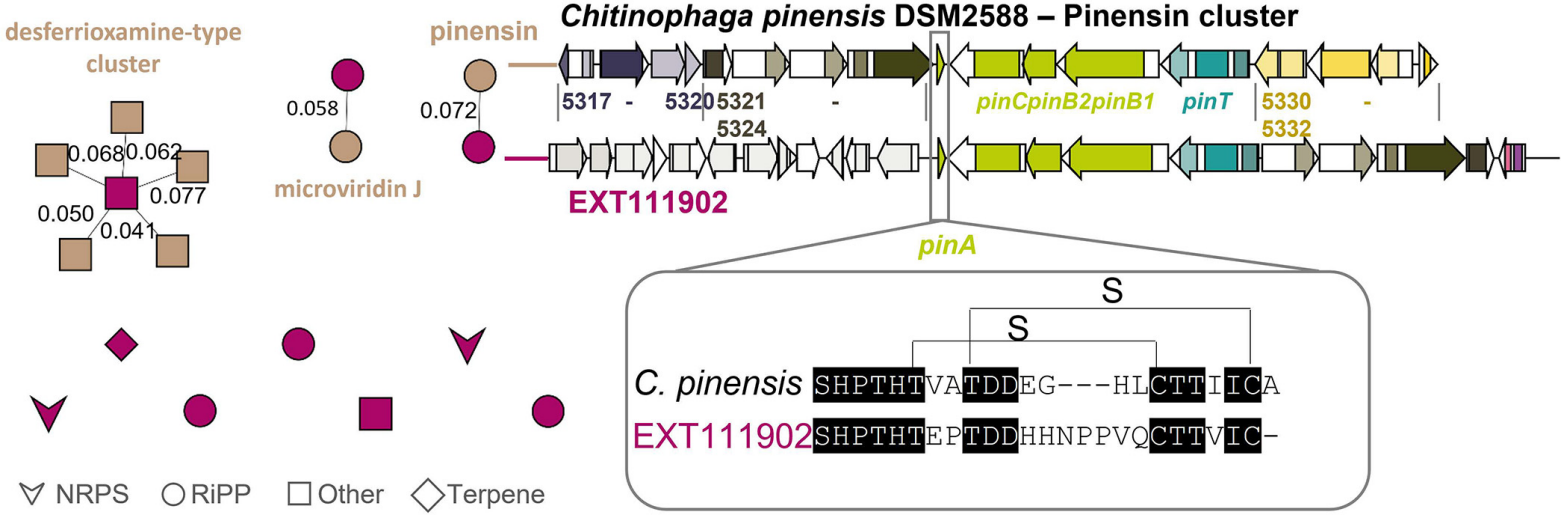
BiG-SCAPE network (16) (left) of strain EXT111902 (magenta) (manually curated). EXT111902 carries five ribosomally synthesized and posttranslationally modified peptides (RiPPs; circle), two nonribosomal peptides (NRPSs; arrow), one terpene (diamond), and two other BGCs (rectangle). By networking with the MIBiG reference clusters (brown), a desferrioxamine-like NRPS-independent siderophore BGC (similarity index up to 0.077, BGC0001478), a microviridin-like BGC (similarity index of 0.058, BGC0000593), and a pinensin-like BGC (similarity index of 0.072) were annotated. Alignment of the pinensin-like cluster of EXT11902 to the pinensin BGC0001392 of *C. pinensis* DSM2588 (right) shows the presence of homologous genes to the biosynthetic core genes *pinA* to *C* (light green) and *pinT* (turquoise), including the split dehydratase genes *pinB1* and *pinB2*. Homologous genes to Cpin5321 to 5324 (dark green) of BGC0001392, suggested to be involved in the perception and import of pinensin, are present. Homologs to Cpin5330 to 5332 (yellow), proposed to be responsible for pinensin export, instead are not detected in the EXT111902 BGC. Alignment of the pinensin core peptide (bottom right) suggests a sequence of 24 amino acids for EXT111902, while the pinensin A peptide contains 22 amino acids.

antiSMASH v6.0 (14) was employed to predict the biosynthetic gene clusters (BGCs). BGC annotation was achieved by their clustering with MIBiG (15) reference clusters into gene cluster families (GCFs) using BiG-SCAPE (16) and setting a cutoff value of 0.6. EXT111902 carries one BGC clustering with BGC0001478, encoding the synthesis of the siderophore desferrioxamine E (17) and another BGC was annotated to BGC0000593, encoding microviridin J (18). The third database annotation refers to pinensin (BGC0001392) (4). Pinensin-like BGCs were detected previously in the genomes of certain *Chitinophaga*, *Chryseobacterium*, *Elizabethkingia*, *Pedobacter*, and *Sinomicrobium* strains (19). Variations in the amino acid sequence of the core peptides indicate a yet undiscovered structural diversity within this lantipeptide type. Such alterations toward the known pinensins are also predicted for EXT111902 (Fig. 1).

### Data availability.

The whole-genome shotgun project has been deposited at DDBJ/ENA/GenBank under BioProject PRJNA904543 with accession number SAMN31846359. Raw data can be obtained from the Sequence Read Archive (SRP421602). The draft genome sequence has been deposited in GenBank under the accession number JAPPSR000000000.
